# Supplementary material for: Sector of Employment and Mortality: A Cohort Based on Different Administrative Archives
Source: Int J Environ Res Public Health. 2023 May 9;20(10):5767. doi: 10.3390/ijerph20105767 (PMC10218361; doi:10.3390/ijerph20105767)
Supplement: Supplementary file 1 [file ijerph-20-05767-s001.zip › ijerph-2316038-supplementary.pdf]

Supplemental Material

Sector of employment and mortality: a cohort based on different administrative archives

Lisa Bauleo [1], Stefania Massari [2], Claudio Gariazzo [2], Paola Michelozzi [1], Luca Dei Bardi [1,3], Nicolas Zengarini [4], Sara Maio [5], Massimo Stafoggia [1], Marina Davoli [1], Giovanni Viegi [5], Alessandro Marinaccio [2] and Giulia Cesaroni [1,*] on behalf of the BIGEPI Collaborative Group.

[1] Department of Epidemiology – Lazio Regional Health Service, ASL Roma 1, Rome, Italy; l.bauleo@deplazio.it, p.michelozzi@deplazio.it; l.deibardi@deplazio.it; m.stafoggia@deplazio.it; m.davoli@deplazio.it; g.cesaroni@deplazio.it .
[2] Italian National Institute for Insurance against Accidents at Work (Inail), Department of Occupational and Environmental Medicine, Epidemiology and Hygiene, Rome, Italy.; s.massari@inail.it; c.gariazzo@inail.it; a.marinaccio@inail.it .
[3] Sapienza University of Rome; Italy.
[4] Regional Public Health Observatory (SEPI), ASL TO3; Grugliasco (TO), Italy; nicolas.zengarini@epi.piemonte.it
[5] Institute of Clinical Physiology, CNR, Pisa, Italy; sara.maio@cnr.it; viegig@ifc.cnr.it.

* Correspondence: g.cesaroni@deplazio.it; Tel.: +39-0699722183

Table S1. Characteristics of the population by sector of employment. Men and women, Rome 2011

Employment sector	Men								Women							
	N*	age (m, sd)		% low education	% high education	% born in	% born abroad	% married	N*	age (m, sd)		% low education	% high education	% born in Rome	% born abroad	% married
**Total**	478,199	54	14	41	18	59	7	71	432,360	51	13	34	19	60	6	71
**Agriculture, forestry and fishing**	22,595	66	13	74	7	21	4	83	28,599	67	12	83	4	13	1	83
**Industry**																
Steel industry	72,872	50	12	35	23	66	4	71	29,807	46	10	14	31	68	4	71
Printing and publishing	17,140	57	13	50	11	68	2	76	11,573	53	12	34	16	70	3	76
Pharmaceutical and chemical industry	17,438	56	13	25	34	56	3	78	11,161	52	13	20	31	61	5	78
Manufacturing	13,737	55	13	29	26	63	4	76	8,559	51	11	21	22	66	4	76
Manufacture of textiles	3,939	56	14	63	7	51	12	76	10,926	54	12	71	4	50	6	76
Electricity, gas, steam and air conditioning su	6,340	56	14	26	31	60	2	77	3,124	51	13	15	34	64	3	77
Food and tobacco production	12,079	54	14	59	10	56	8	76	6,857	48	12	49	11	63	7	76
Non-metal mineral industry	1,066	60	14	57	12	46	5	85	337	56	12	16	11	63	5	85
Glass-ceramic	1,216	55	14	63	8	59	4	78	432	53	11	43	9	51	4	78
Metal processing	605	50	11	65	6	63	7	75	117	48	11	43	12	56	3	75
Manufacture of electrical equipment	27,682	54	12	26	24	63	3	77	10,389	53	12	32	22	59	4	77
Footwear and wood	7,736	56	14	72	4	55	7	78	2,928	51	11	53	8	56	5	78
**Constructions**	74,620	54	15	57	8	53	11	75	14,422	49	11	15	15	70	4	75
**Sales and Transport**										0						
Wholesale and retail trade	98,129	51	13	42	12	66	6	69	105,004	48	11	32	12	71	5	69
Hotels, camping, bars, restaurants	38,336	49	14	54	7	46	20	64	28,989	48	13	47	9	55	15	64
Transportation and storage	64,579	54	14	46	12	85	5	73	19,673	48	12	14	25	93	8	73
**Credit, insurances and services**																
Insurance activities	84,831	51	14	20	28	66	4	66	90,992	46	11	15	24	70	5	66
Healthcare	9,008	52	13	24	38	60	6	70	29,671	50	13	31	19	58	8	70
Services	71,231	57	14	33	29	53	6	74	112,747	55	13	28	28	55	6	74
**Other services**																
Washing and dry-cleaning of textile	1,367	52	13	50	15	61	7	75	2,161	51	12	71	4	58	7	75
Waste collection, treatment and disposal acti	2,070	46	12	54	8	76	4	59	998	44	10	37	10	78	2	59
Hairdressing, salons	1,768	50	14	80	2	57	5	60	7,747	45	10	80	2	73	4	60
Cleaning services	14,888	48	12	63	5	65	12	62	33,270	52	13	72	3	62	9	62
Gas stations	2,360	49	13	68	5	53	17	72	426	44	10	31	11	70	8	72

* the sum of subjects cross the sectors is greater than the total popolation becouse each persons can work in more than sector.

Table S2. Characteristics of the study population by lifetime prevalent sector of employment. Men and women, Rome 2011

	Men								Women							
Employment sector	N	age (mean, sd)		% low education	% high education	% born in Rome	% born abroad	% married	N	age (mean, sd)		% low education	% high education	% born in Rome	% born abroad	% married
**Total**	478,199	54	14	41	19	59	7	71	432,360	52	13	34	21	60	6	63
**Agriculture, forestry and fishing**	11,810	66	14	71	9	25	5	80	23,588	69	11	85	4	13	1	68
**Industry**																
Steel industry	44,505	50	13	34	24	66	4	68	18,507	46	10	13	36	67	4	64
Printing and publishing	13,014	58	14	53	11	68	2	77	8,379	54	13	37	18	71	2	64
Pharmaceutical and chemical industry	12,123	58	14	24	37	55	4	78	8,041	54	13	21	35	61	5	63
Manufacturing	6,763	60	14	29	28	63	5	73	4,671	53	13	23	24	66	4	63
Manufacture of textiles	2,249	58	15	67	6	48	15	74	7,738	56	13	75	4	49	6	71
Electricity, gas, steam and air conditioning su	5,547	58	15	28	28	60	2	78	2,666	52	13	16	33	64	3	62
Food and tobacco production	7,653	55	15	59	11	56	8	76	4,211	49	13	50	12	62	8	66
Non-metal mineral industry	514	61	15	53	14	47	8	84	189	59	13	20	10	61	5	63
Glass-ceramic	700	57	15	65	7	62	5	76	263	55	12	44	9	50	4	79
Metal processing	285	49	12	65	7	69	9	72	71	48	11	41	15	58	3	77
Manufacture of electrical equipment	15,908	56	13	26	24	62	3	77	6,597	57	13	39	18	58	5	66
Footwear and wood	4,551	58	15	74	4	53	8	77	1,722	52	11	57	8	54	5	74
**Construction**	52,837	55	16	58	8	50	14	74	8,616	50	12	16	16	69	5	65
**Sales and Transport**																
Wholesale and retail trade	65,687	51	14	43	12	67	7	67	78,107	48	12	34	13	70	6	64
Hotels, camping, bars, restaurants	28,250	50	14	54	9	42	24	63	18,967	49	14	47	11	51	19	55
Transportation and storage	68,160	56	14	43	13	64	4	75	21,531	49	13	12	28	67	6	58
**Credit, insurances and services**																
Insurance activities	60,140	52	14	18	30	65	4	66	64,411	47	12	14	27	70	5	59
Healthcare	7,528	52	13	22	50	60	7	70	24,801	51	13	31	30	57	9	62
Services	55,984	59	14	31	33	51	6	74	94,417	57	13	27	32	53	6	66
**Other services**																
Washing and dry-cleaning of textile	833	54	14	48	19	59	7	74	1,598	52	12	74	5	56	8	71
Waste collection, treatment and disposal acti	1,185	45	13	55	8	79	4	51	655	42	8	37	11	81	2	59
Hairdressing, salons	1,568	51	14	81	2	56	4	60	6,249	46	10	81	3	73	4	69
Cleaning services	9,149	49	12	63	5	64	14	59	26,203	53	13	73	4	61	10	64
Gas stations	1,256	50	14	68	6	47	23	70	162	44	10	22	14	72	7	57

Table S3. Duration of lifetime prevalent employment (in months). Men and Women, Rome 2011

	Men									Women								
	Mean	Std Dev	5th Pctl	25th Pctl	50th Pctl	75th Pctl	95th Pctl	Min	Max	Mean	Std Dev	5th Pctl	25th Pctl	50th Pctl	75th Pctl	95th Pctl	Min	Max
**Agriculture, forestry and fishing**	130	129	24	45	73	170	431	13	658	105	96	24	48	68	129	322	13	657
**Industry**																		
Steel industry	138	95	22	62	118	193	327	13	459	135	92	23	61	117	185	310	13	455
Printing and publishing	192	118	24	86	188	291	392	13	451	152	113	20	54	122	238	367	13	453
Pharmaceutical and chemical industry	179	108	25	90	167	260	370	13	454	161	103	24	77	142	236	355	13	454
Manufacturing	131	92	19	54	111	194	304	13	460	128	93	19	51	105	192	304	13	451
Manufacture of textiles	106	85	17	38	82	152	288	13	436	91	77	16	34	67	122	250	13	452
Electricity, gas, steam and air conditioning supply	257	131	43	130	274	368	438	13	548	241	140	37	109	253	371	443	13	555
Food and tobacco production	152	104	21	64	128	228	347	13	447	106	92	17	37	77	142	307	13	450
Non-metal mineral industry	142	87	24	69	132	202	296	13	405	134	72	24	70	134	205	234	15	276
Glass-ceramic	135	99	20	54	110	208	323	13	416	103	95	15	32	68	142	314	13	439
Metal processing	128	95	21	55	108	172	341	13	419	93	74	19	38	73	111	237	14	377
Manufacture of electrical equipment	180	107	27	92	166	257	372	13	453	148	95	24	77	129	207	342	13	452
Footwear and wood	123	95	18	44	97	181	315	13	453	80	68	16	31	59	110	226	13	414
**Construction**	128	97	18	48	101	189	320	13	454	122	95	18	47	94	176	311	13	458
**Sales and Transport**																		
Wholesale and retail trade	139	102	20	55	113	203	345	13	459	122	97	18	46	94	170	329	13	460
Hotels, camping, bars, restaurants	133	103	18	48	104	193	341	13	456	97	80	16	35	71	134	267	13	451
Transportation and storage	216	128	31	102	211	332	421	13	537	186	119	29	90	156	269	412	13	548
**Credit, insurances and services**																		
Insurance activities	188	132	22	66	159	303	411	13	558	139	116	19	46	99	207	386	13	507
Healthcare	166	121	20	59	135	261	388	13	453	150	114	19	55	116	228	381	13	454
Services	162	127	17	46	131	262	399	13	456	128	115	16	36	83	199	375	13	459
**Other services**																		
Washing and dry-cleaning of textile	156	107	20	61	141	242	361	13	450	90	77	16	32	65	122	256	13	434
Waste collection, treatment and disposal activities	90	48	24	59	87	118	183	13	320	86	38	25	60	79	118	122	13	275
Hairdressing, salons	72	64	17	29	49	90	211	13	432	75	64	16	30	54	97	211	13	444
Cleaning services	120	88	18	47	98	177	296	13	439	112	77	19	48	95	159	258	13	441
Gas stations	109	84	17	43	88	152	281	13	425	85	77	15	28	61	118	245	13	398

Table S4A. Lifetime prevalent sector of employment and non-accidental and accidental mortality. Men, Rome 2011-2019

	N	Person years	Non accidental mortality: deaths	Crude mortality rates	Age-standardized mortality rates (per 10,000)	95% CI		Accidental mortality: deaths	Crude mortality rates	Age-standardized mortality rates (per 10,000)	95% CI	
**Agriculture, forestry and fishing**	11,810	74,238	2244	302	819	776	864	91	12	40	31	52
**Industry**												
Steel industry	44,505	301,881	2214	73	820	777	866	130	4	40	32	52
Printing and publishing	13,014	85,932	1420	165	846	794	902	53	6	36	26	50
Pharmaceutical and chemical industry	12,123	79,876	1194	149	736	689	787	57	7	46	32	66
Manufacturing	6,763	44,836	659	147	901	822	989	19	4	23	14	36
Manufacture of textiles	2,249	14,595	251	172	893	768	1038	12	8	46	24	89
Electricity, gas, steam and air conditioning supply	5,547	36,850	572	155	748	682	820	19	5	29	16	51
Food and tobacco production	7,653	50,250	810	161	944	867	1027	41	8	48	33	68
Non-metal mineral industry	514	3,365	75	223	714	561	910	3	9	86	20	376
Glass-ceramic	700	4,514	82	182	976	742	1285	3	7	35	11	110
Metal processing	285	1,891	21	111	1093	619	1930	1	5	33	5	233
Manufacture of electrical equipment	15,908	107,336	1205	112	749	697	805	55	5	43	28	67
Footwear and wood	4,551	29,678	576	194	966	874	1068	12	4	23	11	48
**Construction**	52,837	346,164	5921	171	923	896	951	251	7	41	35	48
**Sales and Transport**												
Wholesale and retail trade	65,687	441,432	4,142	94	816	786	847	188	4	37	31	45
Hotels, camping, bars, restaurants	28,250	187,597	1,947	104	914	864	967	87	5	34	27	44
Transportation and storage	68,160	456,417	5,754	126	766	738	794	224	5	34	27	42
**Credit, insurances and services**												
Insurance activities	60,140	404,576	3818	94	765	734	797	179	4	35	30	43
Healthcare	7,528	50,962	457	90	913	799	1042	19	4	39	22	69
Services	55,984	369,432	6111	165	766	743	789	265	7	38	33	44
**Other services**												
Washing and dry-cleaning of textile	833	5,514	60	109	811	603	1092	1	2	11	2	76
Waste collection, treatment and disposal activities	1,185	8,096	49	61	704	498	994	6	7	22	10	49
Hairdressing, salons	1,568	10,578	85	80	824	627	1083	6	6	105	43	258
Cleaning services	9,149	61,532	548	89	1084	973	1209	24	4	23	14	37
Gas stations	1,256	8,395	63	75	643	489	845	5	6	34	14	87

Table S4B. Lifetime prevalent sector of employment and non-accidental and accidental mortality. Women, Rome 2011-2019

	N	Person years	Non accidental deaths: deaths	Crude mortality rate	Age-standardized mortality rates (per 10,000)	95% CI		Accidental deaths: deaths	Crude mortality rate	Age-standardized mortality rates (per 10,000)	95% CI	
**Agriculture, forestry and fishing**	28,599	155,037	3190	206	516	542	491	133	9	22	16	29
**Industry**												
Steel industry	29,807	128,732	311	24	574	704	469	21	2	32	16	61
Printing and publishing	11,573	57,458	418	73	562	632	499	18	3	24	15	40
Pharmaceutical and chemical industry	11,161	55,288	360	65	477	539	423	19	3	27	17	43
Manufacturing	8,559	32,162	195	61	502	594	423	8	2	28	14	58
Manufacture of textiles	10,926	53,091	383	72	460	518	408	22	4	26	16	41
Electricity, gas, steam and air conditioning supp	3,124	18,427	114	62	578	753	444	2	1	5	1	21
Food and tobacco production	6,857	29,037	135	46	498	608	408	8	3	25	11	58
Non-metal mineral industry	337	1,300	9	69	333	750	148	0	0	0	0	0
Glass-ceramic	432	1,789	12	67	542	1,089	269	1	6	69	10	490
Metal processing	117	497	1	20	82	582	12	0	0	0	0	0
Manufacture of electrical equipment	10,389	45,301	356	79	528	607	460	15	3	31	18	54
Footwear and wood	2,928	11,852	54	46	677	1,176	390	4	3	68	20	235
**Construction**	14,422	59,575	278	47	511	597	437	16	3	32	19	54
**Sales and Transport**												
Wholesale and retail trade	105,004	539,938	1895	35	478	511	447	85	2	25	18	37
Hotels, camping, bars, restaurants	28,989	128,478	774	60	582	632	537	22	2	26	13	52
Transportation and storage	19,673	148,896	620	42	518	594	451	27	2	26	17	42
**Credit, insurances and services**												
Insurance activities	90,992	446,554	1490	33	522	561	486	60	1	26	18	37
Healthcare	29,671	170,143	1010	59	533	575	495	48	3	33	21	50
Services	112,747	645,419	5673	88	521	538	505	234	4	24	20	28
**Other services**												
Washing and dry-cleaning of textile	2,161	11,055	59	53	547	771	389	1	1	9	1	65
Waste collection, treatment and disposal activit	998	4,562	6	13	113	416	30	0	0	0	0	0
Hairdressing, salons	7,747	43,507	98	23	497	701	353	7	2	42	16	111
Cleaning services	33,270	178,552	1478	83	621	661	582	58	3	25	17	37
Gas stations	426	1,097	3	27	237	964	58	0	0	0	0	0

Table S5A. Association between lifetime prevalent sector of employment and non-accidental and accidental mortality. Men, Rome 2011-2019.

	Non-accidental mortality							Accidental mortality						
	deaths	age-adjusted HR (95%CI)			fully-adjusted HR (95%CI)			deaths	age-adjusted HR (95%CI)			fully-adjusted HR (95%CI)		
**Agriculture, forestry and fishing**	2244	1.09	1.03	1.15	1.02	0.96	1.07	91	1.12	0.87	1.44	1.02	0.78	1.33
**Industry**														
Steel industry	2214	1.09	1.03	1.14	1.00	0.95	1.06	130	1.19	0.95	1.50	1.10	0.88	1.39
Printing and publishing	1420	1.14	1.08	1.22	1.01	0.95	1.08	53	1.02	0.75	1.39	0.89	0.66	1.22
Pharmaceutical and chemical industry	1194	1.00	0.93	1.06	0.99	0.93	1.06	57	1.11	0.83	1.50	1.14	0.84	1.54
Manufacturing	659	1.16	1.07	1.26	1.11	1.02	1.20	19	0.75	0.47	1.20	0.69	0.43	1.11
Manufacture of textiles	251	1.16	1.02	1.32	1.03	0.91	1.17	12	1.31	0.73	2.35	1.11	0.62	2.01
Electricity, gas, steam and air conditioning supply	572	0.99	0.91	1.09	0.94	0.86	1.02	19	0.75	0.47	1.21	0.73	0.45	1.17
Food and tobacco production	810	1.27	1.17	1.37	1.15	1.07	1.24	41	1.46	1.04	2.05	1.29	0.91	1.81
Non-metal mineral industry	75	1.04	0.82	1.30	0.99	0.79	1.25	3	0.98	0.31	3.08	0.92	0.29	2.91
Glass-ceramic	82	1.23	0.99	1.53	1.09	0.88	1.36	3	1.03	0.33	3.23	0.90	0.29	2.81
Metal processing	21	2.12	1.38	3.25	1.75	1.14	2.69	1	1.79	0.25	####	1.43	0.20	10.22
Manufacture of electrical equipment	1205	1.00	0.93	1.06	0.95	0.89	1.02	55	1.05	0.78	1.42	1.02	0.75	1.38
Footwear and wood	576	1.32	1.21	1.44	1.15	1.05	1.26	12	0.66	0.37	1.18	0.56	0.31	1.00
**Construction**	5921	1.23	1.18	1.28	1.12	1.08	1.17	251	1.16	0.95	1.40	1.01	0.83	1.24
**Sales and Transport**														
Wholesale and retail trade	4,142	1.14	1.09	1.19	1.03	0.99	1.08	188	1.04	0.85	1.28	0.92	0.74	1.13
Hotels, camping, bars, restaurants	1,947	1.25	1.18	1.32	1.12	1.06	1.19	87	1.10	0.85	1.43	0.94	0.72	1.23
Transportation and storage	5,754	1.07	1.03	1.11	0.98	0.94	1.02	224	0.98	0.81	1.20	0.91	0.74	1.11
**Credit, insurances and services**														
Insurance activities	3818	1.00			1.00			179	1.00			1.00		
Healthcare	457	1.12	1.02	1.24	1.15	1.04	1.27	19	0.95	0.59	1.53	0.93	0.58	1.50
Services	6111	1.02	0.98	1.06	1.01	0.96	1.05	265	1.03	0.85	1.25	0.97	0.80	1.17
**Other services**														
Washing and dry-cleaning of textile	60	1.08	0.84	1.40	1.02	0.79	1.32	1	0.39	0.06	2.82	0.36	0.05	2.58
Waste collection, treatment and disposal activities	49	1.10	0.83	1.46	0.95	0.72	1.26	6	2.19	0.97	4.93	1.73	0.76	3.91
Hairdressing, salons	85	1.04	0.84	1.29	0.86	0.69	1.07	6	1.45	0.64	3.28	1.11	0.49	2.52
Cleaning services	548	1.51	1.38	1.66	1.27	1.16	1.39	24	1.16	0.76	1.78	0.91	0.59	1.40
Gas stations	63	0.90	0.70	1.16	0.80	0.63	1.03	5	1.40	0.58	3.42	1.19	0.49	2.90

Fully-adjusted models: adjusted for age, marital status, education, place of birth

Table S5B. Association between lifetime prevalent sector of employment and non-accidental and accidental mortality. Women , Rome 2011-2019.

	Non-accidental mortality							Accidental mortality						
	deaths	age-adjusted HR (95%CI)			fully-adjusted HR (95%CI)			deaths	age-adjusted HR (95%CI)			fully-adjusted HR (95%CI)		
**Agriculture, forestry and fishing**	3190	1.01	0.94	1.08	1.01	0.94	1.08	133	0.97	0.71	1.33	1.03	0.74	1.45
**Industry**														
Steel industry	311	0.97	0.86	1.10	0.97	0.86	1.10	21	1.64	0.99	2.69	1.77	1.07	2.92
Printing and publishing	418	1.02	0.91	1.14	1.02	0.91	1.14	18	1.17	0.69	1.98	1.21	0.71	2.07
Pharmaceutical and chemical industry	360	0.91	0.81	1.03	0.91	0.81	1.03	19	1.21	0.72	2.03	1.23	0.73	2.07
Manufacturing	195	1.01	0.87	1.17	1.01	0.87	1.17	8	1.07	0.51	2.23	1.03	0.49	2.17
Manufacture of textiles	383	0.88	0.78	0.98	0.88	0.78	0.98	22	1.44	0.88	2.36	1.41	0.85	2.33
Electricity, gas, steam and air conditioning supply	114	0.95	0.79	1.16	0.95	0.79	1.16	2	0.42	0.10	1.71	0.46	0.11	1.90
Food and tobacco production	135	1.04	0.87	1.24	1.04	0.87	1.24	8	1.63	0.78	3.41	1.69	0.80	3.55
Non-metal mineral industry	9	0.72	0.37	1.39	0.72	0.37	1.39		0.00	0.00	0.00	0.00	0.00	0.00
Glass-ceramic	12	0.93	0.52	1.64	0.93	0.52	1.64	1	2.12	0.29	15.32	2.08	0.29	15.12
Metal processing	1	0.64	0.09	4.55	0.64	0.09	4.55		0.00	0.00	0.00	0.00	0.00	0.00
Manufacture of electrical equipment	356	1.02	0.91	1.15	1.02	0.91	1.15	15	1.25	0.71	2.21	1.27	0.72	2.26
Footwear and wood	54	1.02	0.78	1.34	1.02	0.78	1.34	4	2.27	0.82	6.24	2.39	0.86	6.63
**Construction**	278	1.01	0.89	1.15	1.01	0.89	1.15	16	1.51	0.87	2.63	1.54	0.88	2.69
**Sales and Transport**														
Wholesale and retail trade	1895	0.95	0.88	1.02	0.95	0.88	1.02	85	1.16	0.83	1.61	1.17	0.84	1.64
Hotels, camping, bars, restaurants	774	1.11	1.02	1.21	1.11	1.02	1.21	22	0.77	0.47	1.26	0.73	0.44	1.21
Transportation and storage	620	1.04	0.94	1.14	1.04	0.94	1.14	27	1.23	0.78	1.93	1.24	0.78	1.95
**Credit, insurances and services**														
Insurance activities	1490	1.00			1.00			60	1.00			1.00		
Healthcare	1010	1.05	0.97	1.14	1.05	0.97	1.14	48	1.27	0.87	1.86	1.28	0.86	1.89
Services	5673	1.00	0.95	1.06	1.00	0.95	1.06	234	1.01	0.76	1.35	1.07	0.80	1.44
**Other services**														
Washing and dry-cleaning of textile	59	0.89	0.69	1.16	0.89	0.69	1.16	1	0.41	0.06	2.96	0.40	0.06	2.93
Waste collection, treatment and disposal activities	6	1.12	0.50	2.51	1.12	0.50	2.51	0	0.00	0.00	0.00	0.00	0.00	0.00
Hairdressing, salons	98	0.94	0.77	1.16	0.94	0.77	1.16	7	1.89	0.86	4.14	1.79	0.80	3.99
Cleaning services	1478	1.20	1.11	1.29	1.20	1.11	1.29	58	1.23	0.86	1.77	1.25	0.85	1.82
Gas stations	3	1.66	0.53	5.17	1.66	0.53	5.17	0	0.00	0.00	0.00	0.00	0.00	0.00

Fully-adjusted models: adjusted for age, marital status, education, place of birth
